# Global cancer research in the era of COVID-19: a bibliometric analysis

**DOI:** 10.3332/ecancer.2021.1264

**Published:** 2021-07-07

**Authors:** Mieke Van Hemelrijck, Grant Lewison, Louis Fox, Verna DNK Vanderpuye, Raúl Murillo, Chris M Booth, Karen Canfell, C S Pramesh, Richard Sullivan, Deborah Mukherij

**Affiliations:** 1 King's College London, Faculty of Life Sciences and Medicine, School of Cancer and Pharmaceutical Sciences, Translational Oncology and Urology Research (TOUR), London SE1 9RT, UK; 2 King's College London, Faculty of Life Sciences and Medicine, Institute of Cancer Policy, London SE1 9RT, UK; 3 National Center for Radiotherapy, Oncology and Nuclear Medicine, Korle Bu Teaching Hospital, PO Box KB369, Accra, Ghana; 4 Hospital Universitario San Ignacio, Colombia; 5 Queens University, Kingston, Ontario, Canada; 6 The Daffodil Centre, The University of Sydney, A Joint Venture with Cancer Council NSW, Australia; 7 Tata Memorial Centre, Homi Bhabha National Institute, Mumbai 400012, India; 8 Naef K. Basile Cancer Institute, American University of Beirut Medical Center, Beirut, Lebanon

**Keywords:** cancer, COVID-19, socio-technical studies, global oncology

## Abstract

**Background:**

Patients with cancer across the world have been impacted by the COVID-19 pandemic due to increased risk of infection and disruption to cancer diagnosis and treatment. Widening of healthcare disparities is expected as the gap between health systems with and without adequate resources to mitigate the pandemic become more apparent. We undertook a bibliometric analysis of research related to cancer and COVID-19 to understand (1) the type of research that has been conducted (e.g. patients, services and systems) and (2) whether the pandemic has impacted the state of global cancer research as measured by research outputs to date.

**Methods:**

An existing filter for cancer research consisting of title words and the names of specialist cancer journals was used to identify cancer and COVID-19 related articles and reviews in the Web of Science (©Clarivate Analytics) between January 2019 and February 2021.

**Results:**

One thousand five hundred and forty-five publications were identified. The majority (57%) were reviews, opinion pieces or concerned with modelling impact of delays to diagnosis and treatment. The main research domains focused on managing or estimating COVID-19 risk to cancer patients accounting for 384 papers (25%). High Income countries contributed the largest volume (*n* = 1,115; 72%), compared to Upper Middle (*n* = 302; 20%), Lower Middle (*n* = 122; 8%) and Low Income countries (*n* = 2.4; 0.2%). No evidence of a reduction in global cancer research output was observed in 2020.

**Conclusions:**

We observed a shift in research focus rather than a decline in absolute output. However, there is variation based on national income and collaborations are minimal. There has been a focus on pan-cancer studies rather than cancer site-specific studies. Strengthening global multidisciplinary research partnerships with teams from diverse backgrounds with regard to gender, clinical expertise and resource setting is essential to prevent the widening of cancer inequalities.

## Introduction

The COVID-19 pandemic has created massive downward pressure on social, economic and health systems across the world. Patients with cancer in all countries and settings have been impacted considerably due to increased morbidity and mortality from COVID-19 infection and delays in cancer therapy [1]. Existing disparities in equitable access to timely and effective cancer care globally are expected to increase as the gap widens between healthcare systems with and without sufficient resources to mitigate the negative effects of the pandemic on cancer prevention, early diagnosis and treatment – as well as access to COVID-19 vaccines [2]. Furthermore, the pandemic has exposed significant deficits in how cancer care and health systems prepare, respond and mitigate such events [3, 4].

Whilst the COVID-19 research and innovation landscape has led to a plethora of review and modelling publications in the context of cancer, the pandemic has exposed significant gaps in our knowledge and conduct of research; from ways to estimate the direct risk to patients with cancer (cancer or modality specific infection and case fatality rates) through to understanding how pandemic-driven public health interventions (non-pharmaceutical interventions) impact the entire cancer pathway from delays to diagnosis to delays/changes in treatment, and palliative care/end-of-life experiences.

Several groups have formed collaborations to collect country- or region-specific data on the impact of COVID-19 on patients with cancer, primarily in high-income countries [5–7]. The COVID-19 and Cancer Global Taskforce (https://covidcancertaskforce.org/) together with the COVID-19 and Cancer Global Modelling Consortium (www.CCGMC.org) has been seeking to understand global COVID and cancer research in order to inform emerging public health priorities. This will be achieved by feeding intelligence from three projects into regional and country research priority co-development processes. The three projects that will feed into this are: 1) a qualitative review about how the pandemic has affected the conduct of clinical cancer research; 2) a snapshot survey, producing original data [8] and 3) a bibliometric analysis.

Here, we report an analysis of research on cancer in the context of SARS-CoV-2/COVID-19 pandemic. We aimed to understand: (1) what COVID-19 (including SARS-CoV-2) research has been conducted related to cancer patients, services and systems and (2) whether the pandemic has impacted the state of global cancer research as measured by research outputs to date.

## Methods

To understand what research on COVID-19 and SARS-CoV-2 virus had been conducted germane to cancer patients, services and systems, we used an existing filter for cancer research, labelled ONCOL, which consisted of title words and the names of specialist cancer journals, to identify articles and reviews in the Web of Science (WoS, ©Clarivate Analytics) within between January 2019 and February 2021 [9]. Within this set, we downloaded papers (as text files including their bibliographic details) with the title words ‘CORONAVIRUS OR SARS OR MERS OR COVID* OR SARS-*’.

For the most recent papers, the publication year was not always given, and was completed with the year of the previous paper in the file, as the WoS lists the papers in reverse chronological order. The addresses on the papers were parsed by a macro to show the fractional count of the countries represented. For example, a paper with one French and two United States addresses would be categorised as FR = 0.33, US = 0.67. The sum of the fractional counts for seven continents was then calculated as defined in Table 1.

Additional macros were used to categorise the papers by their Research Level (RL) on a scale from clinical research = 1.0 to basic research = 4.0 [10], and their focus on individual anatomical sites and research domains [11]. However, one of the most common types of research was reports of the management or the treatment of patients with cancer during the COVID-19 epidemic. These papers were identified by the presence of the following words in their titles: *burnout, consequence, delay, experience, manag..., organis..., organiz..., practic..., practis..., remote, telemedicine, telemonitor.* Many of the other papers were concerned with the comorbidity of patients with cancer who were affected by COVID-19.

We also examined whether the pandemic *per se* had depressed global cancer research (less active research institutions, less funding, etc.). A general cancer filter against the WoS was run for the last 5 years, 2016–20, and identified articles and reviews from the world and from four groups of countries, classified by income level: high (HI), upper middle (UM), lower middle (LM) and low (LO). These classifications are based on income per capita as determined by the World Bank (Appendix A).

## Results

For research that examined COVID-19 (SARS-CoV-2) in the context of any aspect of cancer control and care (patients, systems, etc.), there were 1,545 publications. The tally for 2021 is clearly incomplete and is likely eventually to be higher than the current total for 2020. The mean RL was 1.1, which is very clinical, compared with papers focused on COVID research generally (RL = 1.51) and world cancer (non-COVID) research (RL = 2.10). One hundred and four countries contributed authors to the 1,545 research papers. The countries with the largest contributions are shown in Table 2. Turkey and India (IN) were the only upper middle and low income countries, respectively, with large contributions.

**Figure 1.**
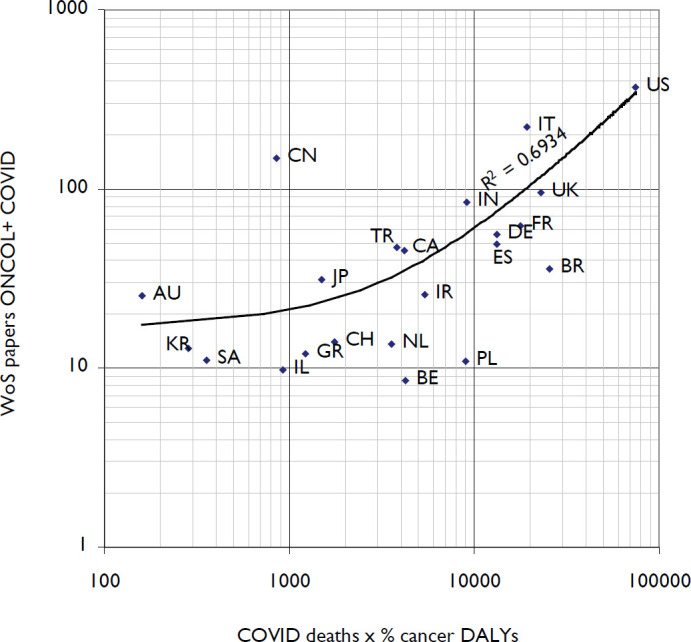
Plot of research output in cancer research plus coronavirus up to February 2021 against number of deaths from COVID-19, multiplied by the percentage of the disease burden from cancer in 2019 for the countries listed in Table 1, plus BE = Belgium, BR = Brazil, GR = Greece, IL = Israel, IR = Iran, JP = Japan, KR = South Korea, NL = Netherlands, PL = Poland, SA = Saudi Arabia.

COVID research germane to cancer control and care was low, ranging from 2% of countries output (China) to a high of 6% (Italy) with an average of 4% of the combined major COVID research focused on cancer. Figure 1 shows the outputs of the different countries compared with the product of the number of deaths from COVID-19 [12] and the percentage of deaths from cancer in 2019 [13]. The correlation (with a linear equation) was positive and quite high. China was a notable outlier, with a large research output but relatively few deaths according to official figures.

**Figure 2.**
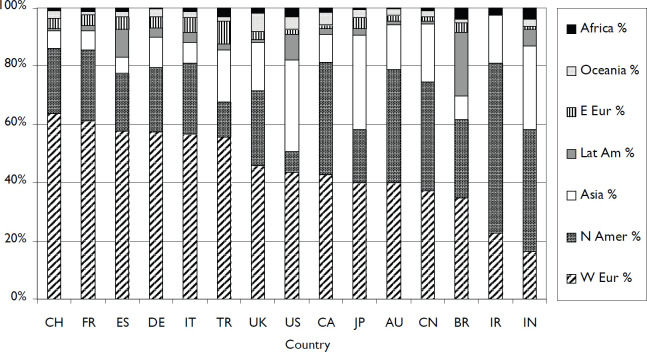
Contributions to the foreign component of the COVID + ONCOL papers of selected countries, from the seven continental regions. Countries are ranked by the percentage contributions from Western Europe.

Figure 2 shows the continental regions with which the leading countries collaborate. However, the numbers of internationally collaborative papers are very small, and the average total foreign contribution to these papers is only 45 per country. There is much more collaboration with Western Europe (45% on average) than with North America (average 28%) except for IN and Iran (IR). Of the ten Iranian papers with international collaboration, US authors contributed to eight. Collaboration from Eastern European, Oceanian and African countries was far less.

**Figure 3.**
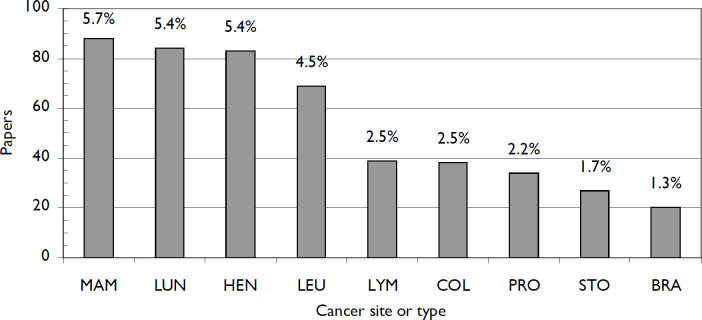
Numbers of ONCOL + COVID papers on different cancer sites and as a % of total ONCOL + COVID output over this period. MAM = breast cancer; LUN = lung cancer; HEN = head & neck cancer; LEU = leukaemia; LYM = lymphoma; COL = colorectal cancer; PRO = prostate cancer; STO = stomach cancer; BRA = brain cancer.

We also examined which site-specific cancers COVID research was focused on. One (or more) was specified for only 543 of the 1,545 papers (35%). In normal times, well over half of cancer research papers are on one or more specified site-specific cancers. The leading ones are as shown in Figure 3.

**Figure 4.**
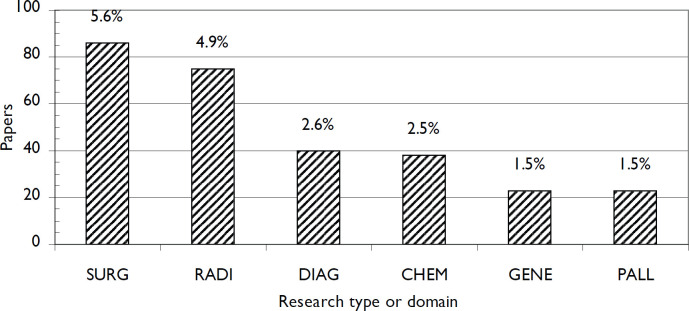
Numbers of ONCOL + COVID papers in different research domains, 2001–21, in the WoS. SURG = surgery; RADI = radiotherapy; DIAG = diagnosis; CHEM = chemotherapy; GENE = genetics; PALL = palliative care.

The main research domain focused on managing or estimating COVID-19 risk to cancer patients. This accounted for 384 papers (25%). The other domains which research focused on were much lower, ranging from 5.6% of the total output (*n* = 1,545) for surgical research to about 1.5% for COVID-cancer research in the palliative care setting (Figure 4). The majority of the research publications (57%) were concerned with reviews, opinion pieces, modelling (impact of delays to diagnosis and treatment) and other specific areas.

Of the 384 papers on the risk of COVID-19 and cancer management, the largest contributions were from the USA (79.8 papers on fractional count) and Italy (64.9 papers). However, as a proportion of the country total, France published the most research in this area (45% of its total output), followed by Canada (36%) and IN (33%). East Asian countries published relatively few papers of their total output on this topic: China 18% and Japan only 14%. High Income countries contributed by far the largest volume of research on this subject (*n* = 1,115; 72%), compared to Upper Middle (*n* = 302; 20%), Lower Middle (*n* = 122; 8%) and Low-income countries (*n* = 2.4; 0.2%).

**Figure 5.**
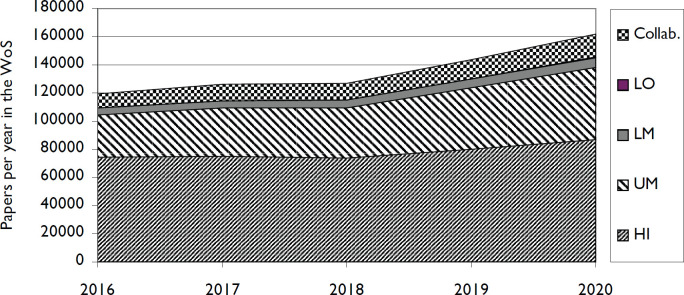
Numbers of cancer research papers from four groups of countries, classified by UN income category. The output from low income countries is too small to appear in this graph, but it increased from 109 in 2019 to 207 in 2020, with no inter-group collaboration, and from 404 to 654 when inter-group collaboration is included.

In seeking to understand whether the pandemic had impacted global cancer research, we also analysed global cancer research trends. The numbers of cancer research papers were determined for each of the four groups including ones written collaboratively with authors from other groups, and for each of the four global income country groups alone. The latter included cancer research papers with intra-group collaboration. A graph of outputs from the second set, plus ones involving inter-group collaboration, is shown in Figure 5. Output from low income countries increased from 109 to 207 (2019 to 2020) with no inter-group collaborations, and from 404 to 654 when inter-group collaborations were included. Subject to the caveats listed in limitations, it appears that there is no evidence of a reduction in global cancer research output in 2020, or by any of the four groups of countries.

There are a number of countries with cancer research outputs where the ratio between outputs in 2020 and 2016 is much greater than the world average of 1.41. Of those with at least 1,000 cancer papers during this period (*n* = 55), the top countries in terms of expansion are shown in Table 3. Low income countries like Egypt, Indonesia and Pakistan were included. This rapid expansion group reflects a diversity of geographies and income levels that have all seen one thing in common, an increasing commitment to cancer research. As such, they are essentially more dynamic compared to the UK, which only saw a ×1.23 growth during the same period and the USA (×1.22 growth). Our analysis shows that the pandemic has had no measurable impact on these countries rate of increase during the period of our analysis.

## Discussion

Our bibliometric analysis did not find any impact to date, subject to the limitations described below, on the volume of global cancer research activity. However, a shift in focus with respect to research domain and cancer type(s) was observed. Finally, the contribution by low or middle income countries was <10% of publications identified.

Cancer is one of the most dominant medical research ecosystems in the modern world. It may be that even with the extended periods of shutdown of laboratories, trials as well as reductions in funding, there will be no noticeable impact on total global research because systems recover quickly and/or one country’s reduced output is made up for by increases elsewhere, e.g. China’s rapid expansion. Some high-income research ecosystems may paradoxically, be relatively over-funded and their furlough systems may have moved people into COVID-19 specific research work rather than generic cancer research. Counterintuitively, reductions in funding may actually lead to increased levels of publications as environments become more competitive.

However, taking into account lag periods for impacts on reduced funding, it may take up to 5 years to demonstrate an impact through reduced outputs as momentum and active grants will continue to feed the system. Moreover, it has been estimated that upcoming funding streams will be reduced.

An important consideration of the effect of the pandemic with regard to academic output is the increased pressure on researchers of both genders, but particularly for women for whom lockdowns and school closures have led to additional childcare responsibilities. Bibliometric analysis of general COVID-19 related publications have suggested fewer publications than expected from female academics [14, 15]. It is vital that the gendered impact of the pandemic is not forgotten since without acknowledging and addressing this, existing disparities will inevitably widen [16, 17]. Impactful scientific investigation cannot occur without research teams that are diverse in all aspects.

Our analysis suggests that ‘bellweather’ countries with the most rapid expansion in cancer research outputs, reflecting a wide range of geographies and income levels, are the most susceptible to downturns and provide the best group for ‘early warning’ from a bibliometrics perspective. Furthermore, for those emerging countries in cancer research in Low and Middle Income Countries (LMICs), the pandemic is likely to have substantial detrimental effects, i.e. stifle any output increases in the next decade without efforts to mitigate this widening of inequity.

Turning to research examining the impact of COVID-19 on patients with cancer, services and systems, we found that relative to the impact of the pandemic and the risk of this virus there was a low level of research activity, and this was heavily dominated by high income countries – potentially also a reflection of difficulties with national data collection in low and middle income countries. However, both IN and China were notable exceptions to this trend. This tilt in research activity was further reflected in the tendency for collaboration(s) with Western Europe. Nevertheless, despite the global impact of the pandemic, it was notable that most research remained essentially ‘national’ with few comparative studies. Most research was pan-cancer, rather than looking at the impact from a site-specific perspective. Again, an impact that may only become apparent in several years from now, as specificities related to specific tumour types emerge. Hence, there is a need for global data science partnerships to allow people to look at the granularity of COVID-19 impact based on cancer type and treatment.

In terms of domain focus, it is unsurprising that the majority of research was focused on the risk of COVID-19 (and its mitigation) to cancer patients, with a relatively low level of focus on specific modalities such as radiotherapy and surgery. Moreover, few studies have focused on the intersection between systemic anti-cancer treatments (SACT) and COVID-19 – which is surprising given that the immune system may be severely affected by both SACT and COVID-19. Even worse, the impact of COVID-19 on palliative care has almost been totally ignored. Finally, the indirect impact of COVID-19 (e.g. lockdowns, economic decline) on services and systems was also very neglected. Moreover, it needs to be noted that early published data on COVID-19 and cancer may suffer from methodological flaws that limit the quality of the evidence [18, 19].

Whilst this analysis shows a detailed assessment of what has been published on COVID-19 and cancer to date, the tally of papers for 2020 will not be complete yet so our analysis will underestimate outputs. Papers will be added to the WoS and will be included in the 2020 tally that may appear in print in 2021. For previous years, the date is the print date. So, the current 2020 tally will be both too low (see above) and also too high because it will include some ‘advance’ papers that are visible now online, but have not yet been printed and may have a print date of 2021. Many of the papers in ‘2020 to 2021’ do not have a publication month, or indeed publication year, so it is hard to know which year to classify them as. It also needs to be noted that a bibliometric analysis is not a full research ecosystem analysis as such, because it does not consider inputs (funding) and projects in process (e.g. continuation or activation of clinical trials).

## Conclusion

The current bibliometric analysis of research output for COVID-19 and cancer shows a shift in research focus rather than a decline in absolute output. However, there is variation based on national income and collaborations are minimal. There has been a focus on pan-cancer studies rather than cancer site-specific studies. Strengthening global multidisciplinary research partnerships with teams from diverse backgrounds with regard to gender, clinical expertise and resource setting is essential to prevent the widening of cancer inequalities in LMICs.

## Conflicts of interest

The authors declare no conflicts of interest.

## Funding statement

This publication is funded through the UK Research and Innovation GCRF RESEARCH FOR HEALTH IN CONFLICT (R4HC-MENA); developing capability, partnerships and research in the Middle and Near East (MENA) ES/P010962/1.

## Figures and Tables

**Table 1.** Continental codes and countries used for analysis.

Code	Continent
AFR	Africa
ASI	Asia
EEU	Eastern Europe (incl. Russia)
EUR	Western Europe
LAT	Latin America and the Caribbean
NAM	North America (Canada and the USA)
OCE	Oceania (Australia & New Zealand)

**Table 2.** Outputs of papers on COVID by the leading 12 countries (*N*), COVID research in cancer (C + O) (*N*) and percentage of these papers, integer counts, as a proportion of countries overall COVID research output over this period.

Country	ISO	COVID	C+O	%
USA	US	15,418	499	3.2
Italy	IT	5,072	298	5.9
UK	UK	5,148	188	3.7
China	CN	8,725	180	2.1
France	FR	2,147	112	5.2
Germany	DE	2,822	110	3.9
Spain	ES	2,533	102	4.0
India	IN	3,416	100	2.9
Canada	CA	2,503	90	3.6
Switzerland	CH	1,119	62	5.5
Turkey	TR	1,471	60	4.1
Australia	AU	2,059	54	2.6

**Table 3.** Top countries (with at least 1,000 papers published between 2016 and 2020) in terms of research expansion. The world average ratio is 1.41.

Country	Level	Output	Ratio
Jordan	UM	1,056	3.62
Lebanon	UM	1,168	3.22
Indonesia	LM	1,162	2.89
Iran	UM	13,160	2.35
Saudi Arabia	HI	6,076	2.30
Pakistan	LM	3,349	2.30
South Africa	UM	2,150	2.04
Egypt	LM	7,719	1.97
Russia	UM	5,407	1.97
Croatia	HI	1,119	1.89
Colombia	UM	1,422	1.86
China	UM	180,856	1.80
Chile	HI	1,653	1.71
Portugal	HI	4,805	1.65

## Appendix A. World Bank classification of countries by income

The countries in each group are as follows (the names are those used by the WoS):

HIGH INCOME:

CU=(ANDORRA OR ANTIGUA-&-BARBU OR ARUBA OR AUSTRALIA OR AUSTRIA OR BAHAMAS OR BAHRAIN OR BARBADOS OR BELGIUM OR BERMUDA OR BRUNEI OR CANADA OR CAYMAN-ISLANDS OR CHILE OR CROATIA OR CURACAO OR CYPRUS OR CZECH REPUBLIC OR DENMARK OR ENGLAND OR ESTONIA OR FINLAND OR FRANCE OR GERMANY OR GREECE OR GREENLAND OR GRENADA OR HUNGARY OR ICELAND OR IRELAND OR ISRAEL OR ITALY OR JAPAN OR KUWAIT OR LATVIA OR LIECHTENSTEIN OR LITHUANIA OR LUXEMBOURG OR MALTA OR NETHERLANDS OR NEW-CALEDONIA OR NEW-ZEALAND OR NORTH-IRELAND OR NORWAY OR OMAN OR PALAU OR PANAMA OR POLAND OR PORTUGAL OR PUERTO-RICO OR QATAR OR SAN-MARINO OR SAUDI ARABIA OR SCOTLAND OR SEYCHELLES OR SINGAPORE OR SINT-MAARTEN OR SLOVAKIA OR SLOVAK-REPUBL OR SLOVENIA OR SOUTH-KOREA OR SPAIN OR ST-KITTS-NEVIS OR SWEDEN OR SWITZERLAND OR TAIWAN OR TRINID-&-TOBAGO OR TRINID-AND-TOBAGO OR TURKS-CAICOS OR U-ARAB-EMIRATES OR URUGUAY OR USA OR WALES)

UPPER MIDDLE INCOME:

CU=(ALBANIA OR ALGERIA OR ANGUILLA OR ARGENTINA OR ARMENIA OR AZERBAIJAN OR BELARUS OR BELIZE OR BOSNIA-&-HERCEG OR BOTSWANA OR BRAZIL OR BULGARIA OR CHINA OR COLOMBIA OR COOK-ISLANDS OR COSTA-RICA OR CUBA OR DOMINICA OR DOMINICAN-REP OR ECUADOR OR EQUAT-GUINEA OR FIJI OR FRENCH-GUIANA OR GABON OR GEORGIA OR GRENADA OR GUATEMALA OR GUYANA OR IRAN OR IRAQ OR JAMAICA OR JORDAN OR KAZAKHSTAN OR LEBANON OR LIBYA OR MACEDONIA OR MALAYSIA OR MALDIVES OR MARSHALL-ISLAND OR MAURITIUS OR MEXICO OR MONTENEGRO OR MONTSERRAT OR NAMIBIA OR NAURU OR NETH-ANTILLES OR NORTH-MACEDONIA OR PARAGUAY OR PERU OR REUNION OR ROMANIA OR RUSSIA OR SAMOA OR SERBIA OR SERBIA-MONTENEG OR SOUTH-AFRICA OR SRI-LANKA OR ST-LUCIA OR ST-VINCENT OR SURINAME OR THAILAND OR TONGA OR TURKEY OR TURKMENISTAN OR VENEZUELA)

LOWER MIDDLE INCOME:

CU=(ANGOLA OR BANGLADESH OR BHUTAN OR BOLIVIA OR CAMBODIA OR CAMEROON OR CAPE-VERDE OR COMOROS OR CONGO OR COTE-IVOIRE OR DJIBOUTI OR EGYPT OR EL-SALVADOR OR ESWATINI OR GHANA OR HONDURAS OR INDIA OR INDONESIA OR KENYA OR KIRIBATI OR KYRGYZSTAN OR LAOS OR LESOTHO OR MAURITANIA OR MICRONESIA OR MOLDOVA OR MONGOLIA OR MOROCCO OR MYANMAR OR NICARAGUA OR NIGERIA OR PAKISTAN OR PAPUA-N-GUINEA OR PHILIPPINES OR REP-CONGO OR SAO-TOME-&-PRIN* OR SENEGAL OR SOLOMON-ISLANDS OR SUDAN OR SWAZILAND OR TIMOR-LESTE OR TUNISIA OR UKRAINE OR UZBEKISTAN OR VANUATU OR VIETNAM OR ZAMBIA OR ZIMBABWE)

LOW INCOME:

CU=(AFGHANISTAN OR BENIN OR BURKINA-FASO OR BURUNDI OR CENT-AFR-REPUBL OR CHAD OR DEM-REP-CONGO OR ERITREA OR ETHIOPIA OR GAMBIA OR GUINEA OR GUINEA-BISSAU OR HAITI OR LIBERIA OR MADAGASCAR OR MALAWI OR MALI OR MOZAMBIQUE OR NEPAL OR NIGER OR NORTH-KOREA OR RWANDA OR SIERRA-LEONE OR SOMALIA OR SOUTH-SUDAN OR SYRIA OR TAJIKISTAN OR TANZANIA OR TOGO OR UGANDA OR YEMEN)
